# Thiophene-based donor–acceptor co-oligomers by copper-catalyzed 1,3-dipolar cycloaddition

**DOI:** 10.3762/bjoc.8.76

**Published:** 2012-05-03

**Authors:** Stefanie Potratz, Amaresh Mishra, Peter Bäuerle

**Affiliations:** 1 University of Ulm, Institute of Organic Chemistry II and Advanced Materials, Albert-Einstein-Allee 11, 89081 Ulm, Germany

**Keywords:** acetylene, azide, click chemistry, thiophene, triazole

## Abstract

Herein we present a three-component one-pot procedure to synthesize co-oligomers of a donor–acceptor–donor type, in which thiophene moieties work as donor and 1,2,3-triazoles as acceptor units. In this respect, terminally ethynylated (oligo)thiophenes were coupled to halogenated (oligo)thiophenes in the presence of sodium azide and a copper catalyst. Optoelectronic properties of various thiophene-1,2,3-triazole co-oligomers were investigated by UV–vis spectroscopy and cyclic voltammetry. Several co-oligomers were electropolymerized to the corresponding conjugated polymers.

## Introduction

Oligo- and polythiophenes are among the most extensively investigated organic semiconducting materials used in organic electronics [1–3]. In this respect, various oligomers have been developed by using metal-catalyzed Suzuki-, Stille-, Sonogashira-, and Kumada-type cross-coupling reactions [4–9]. On the other hand, the so-called “click chemistry”, originally reported by Sharpless and co-workers in 2001, has played a significant role as a versatile strategy for the rapid and efficient assembly of a pair of functional molecular building blocks under mild reaction conditions [10]. It guarantees reliable synthesis of the desired products in high yield and purity. Click reactions generally involve a Cu(I)-catalyzed version of the Huisgen 1,3-dipolar cycloaddition of terminal acetylenes and azides (CuAAC), to regioselectively yield 1,4-disubstituted 1*H*-1,2,3-triazoles [11–12]. In the meanwhile, this type of click reaction has become very popular and reached high significance in materials synthesis, because high yields and easy to purify products are typically obtained [13–14]. The click chemistry approach was successfully employed to synthesize various oligomers [15–17], catenanes and rotaxanes [18], dendrimers [11,19–20], and polymers [21–23], and was used for DNA labelling [24–25], sensors [26–27], and metal chelates [28–30], due to the mild reaction conditions and compatibility with a variety of functional groups. However, the electronic conjugation through the resulting 1,2,3-triazole rings is weak due to poor electronic communication between the chromophores [21–23,31]. It has also been shown that a 1,2,3-triazole can act as a strong σ-electron donor [26] or as a weak π-electron acceptor [15].

In this study, we aimed at the combination of electron-rich (oligo)thiophenes as donors and electron-deficient 1,2,3-triazole rings as acceptors to conveniently build up novel donor–acceptor co-oligomeric and copolymeric materials by click chemistry. Thereby, the inherent instability of 2-azidothiophene was a problem.

## Results and discussion

**Scheme 1:**
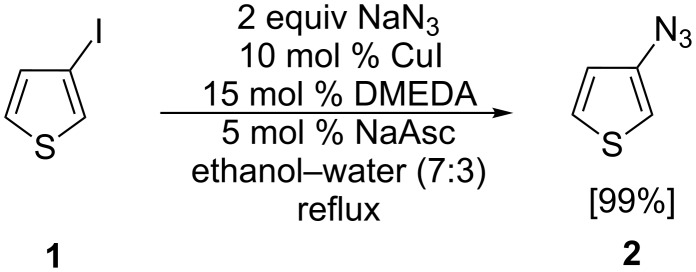
Synthesis of 3-azidothiophene **2**.

In 2005, Liang et al. [32] described a mild, copper-catalyzed method to synthesize aromatic azides from halogenated arenes and sodium azide. We have now transferred this method to the synthesis of 3-azidothiophene (**2**) from 3-iodothiophene (**1**) in excellent yield, which in the following was used for further click reactions to form novel thienyl-1,2,3-triazole co-oligomers (Scheme 1). In contrast, 2-azidothiophene could not be obtained by this protocol, because it is inherently instable. This finding corresponds to the observations of Zanirato et al. that, depending on the nature of the substituents, 5-substituted 2-azidothiophenes are more or less instable at elevated temperatures [33].

**Scheme 2:**
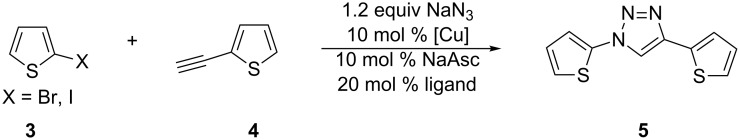
One-pot, two-step procedure to give a dithienyl-1,2,3-triazole co-oligomer **5**. See Table 1 for detailed conditions.

In order to overcome this inherent problem, we used a one-pot, two-step sequence, whereby an organic azide was generated in situ from a corresponding halide and immediately consumed in a reaction with copper acetylide [34–36]. Thus, as a model, we optimized the reaction of 2-halogenothiophene **3** and 2-ethynylthiophene (**4**) in the presence of sodium azide and a copper(I) catalyst to yield co-oligomer 1,4-di(thien-2-yl)-1,2,3-triazole (**5**) (Scheme 2).

We first used the Fokin protocol [34], which worked well for phenyl halides and in which 1.2 equiv of sodium azide, 10 mol % of cupric sulfate, 10 mol % sodium ascorbate, which reduces Cu(II) to Cu(I), 10 mol % sodium carbonate and 20 mol % L-proline as ligand were reacted in the solvent system DMSO–water at 60 °C. The co-oligomeric product **5** was obtained in only 10% yield (Table 1, entry 1). When the stabilizing ligand was changed to *N*,*N*’-dimethylethylenediamine (DMEDA) according to Liang et al. [32] the yield of 1,4-di(thien-2-yl)-1,2,3-triazole (**5**) was increased to 17% (entry 2). Careful choice of solvent mixture and temperature finally raised the yield of **5** to 59% when ethanol–water (7:3) at 50 °C was used (entry 4). Lower temperatures gave an incomplete conversion of 2-iodothiophene (**3**), whereas at 95 °C the 2-azidothiophene eventually decomposed. Therefore, we found that a temperature of 50 °C was a good compromise between the reactivity of the halogenated thiophene in the nucleophilic substitution to 2-azidothiophene, and the stability of the in situ formed azido derivative.

**Table 1:** Variation of solvent mixture and temperature in Cu(I)-catalyzed cycloaddition reactions of 2-iodothiophene (**3**) and 2-ethynylthiophene (**4**) to form co-oligomer **5**.

entry	ligand	solvent	*T* [°C]	yield [%]

1	L-proline	DMSO–water (9:1)	60	10
2	DMEDA	DMSO–water (9:1)	50	17
3	DMEDA	*tert*-butanol–water (2:1)	50	28
4	DMEDA	ethanol–water (7:3)	50	59
5	DMEDA	ethanol–water (7:3)	20	39
6	DMEDA	ethanol–water (7:3)	95	16

Under the optimized reaction conditions for the preparation of di(thien-2-yl)-1,2,3-triazole **5** (2 equiv NaN_3_, 10 mol % copper(I) iodide, 10 mol % sodium ascorbate, 20 mol % DMEDA, ethanol–water (7:3), 50 °C for 15 h, entry 4) a series of conjugated benzene- and thiophene-1,2,3-triazole co-oligomers was synthesized in good to excellent yields (Table 2). By systematic variation of the halogenated and the ethynylated reagent, general trends could be deduced: (a) Iodides react better than bromides (**5**, **6**, **8**, (**9**), **10**); (b) Due to the higher stability of 3- versus 2-azidothiophenes, 3-halogenated thiophenes give higher yields than 2-halogenated thiophenes, 2-substituted benzenes higher yields than thiophenes, and thiophenes higher yields than bithiophenes.

**Table 2:** Synthesis of 1,4-disubstituted 1,2,3-triazoles from corresponding halides (1 equiv), terminal acetylenes (1 equiv) and sodium azide (2 equiv) in the presence of copper(I) iodide (10 mol %), sodium ascorbate (10 mol %) and *N*,*N*’-dimethylethylenediamine (DMEDA, 20 mol %) in ethanol–water (7:3) at 50 °C for 15 h. The yields are for pure compounds and are averages of two runs.

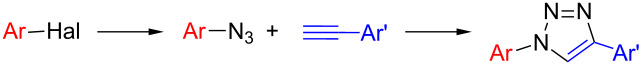					

product	yield [%] (halide)	product	yield [%] (halide)	product	yield [%] (halide)

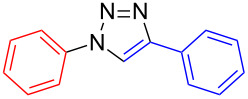 **6**	87 (I) 42 (Br) 99 (I)^a^ 99 (Br)^a,b^	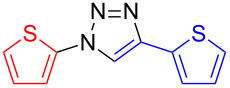 **5**	67 (I) 60 (Br)	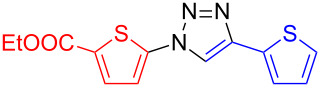 **17**	25 (I) 62 (I)^b^
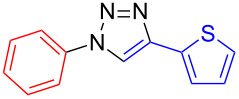 **7**	90 (I)	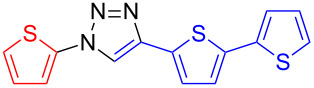 **12**	61 (I)	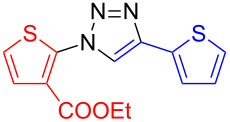 **18**	0 (I)
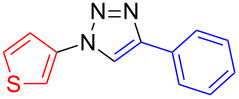 **8**	83 (I) 62 (Br)	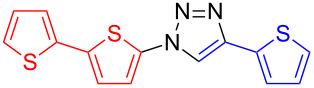 **13**	67 (I)	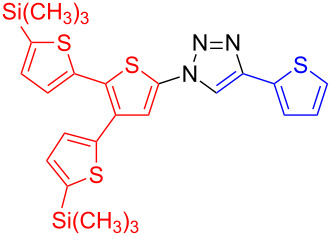 **19**	44 (I)
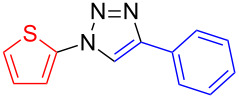 **9**	47 (I) 53 (Br)	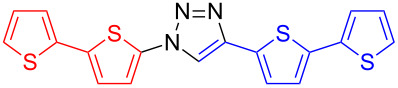 **14**	33 (I)		
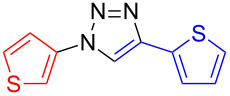 **10**	88 (I) 45 (Br)	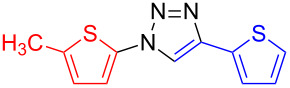 **15**	44 (I) 72 (I)^c^	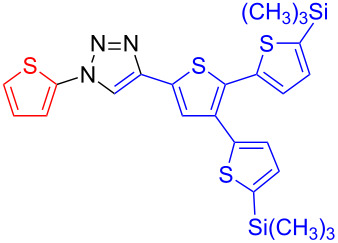 **20**	83 (I)
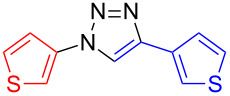 **11**	99 (I)	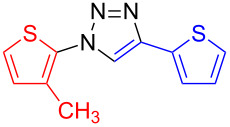 **16**	10 (I)		

^a^Reaction in DMSO–water (9:1); ^b^reaction at 95 °C; ^c^reaction at rt.

As expected, 3-halogenothiophenes gave higher yields than 2-halogenothiophenes (e.g., **8** and **9**), because of the higher nucleophilicity in azide formation. Additionally, 3-azidothiophenes are much more stable than 2-azidothiophenes [37]. In the series of halides, benzenes gave higher yields than mono- and bithiophenes; the lowest conversion was observed for the branched terthiophene to form co-oligomer **19**, which is a result of the instability of the intermediate azide. The electron-donating methyl group in the 5-position of the thiophene ring destabilizes the corresponding azide, therefore low conversion to **15** was observed. The yield was increased with decreasing temperature. Electron-withdrawing ester groups showed an opposite tendency. Due to the lower nucleophilicity of ethyl 5-iodothiophene-2-carboxylate, the conversion to **17** rose with increasing temperature.

**Scheme 3:**
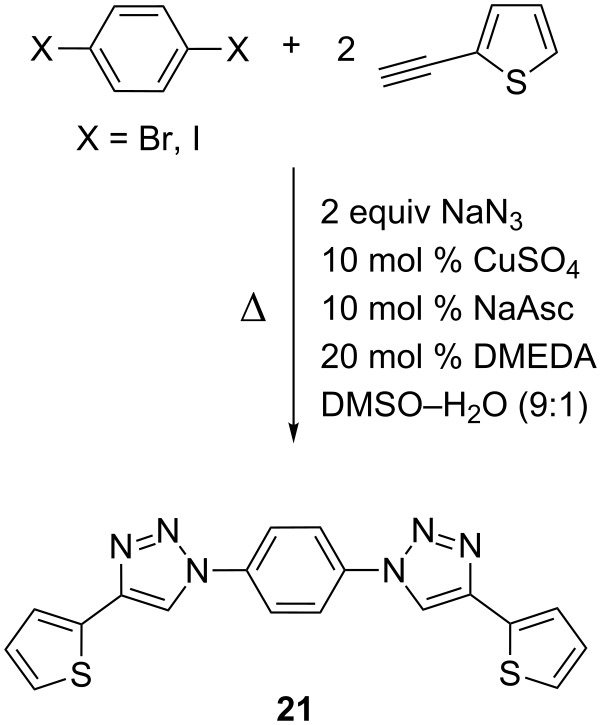
Synthesis of 1,4-bis[4-(thiophen-2-yl)-1*H*-1,2,3-triazol-1-yl]-benzene **21**.

Substituents in the 3-position of 2-iodothiophenes gave low or no conversion to **16** and **18**, respectively, because of steric hindrance in the copper-catalyzed azide formation. Twofold reactions were also investigated under the optimized reaction conditions. 1,4-Dihalogenobenzene was reacted with 2-ethynylthiophene in excellent yields (Scheme 3). 1,4-Diiodobenzene gave the desired product **21** in 99% yield at 50 °C, whereas 1,4-dibromobenzene was disubstituted at 95 °C in 98% yield.

**Figure 1:**
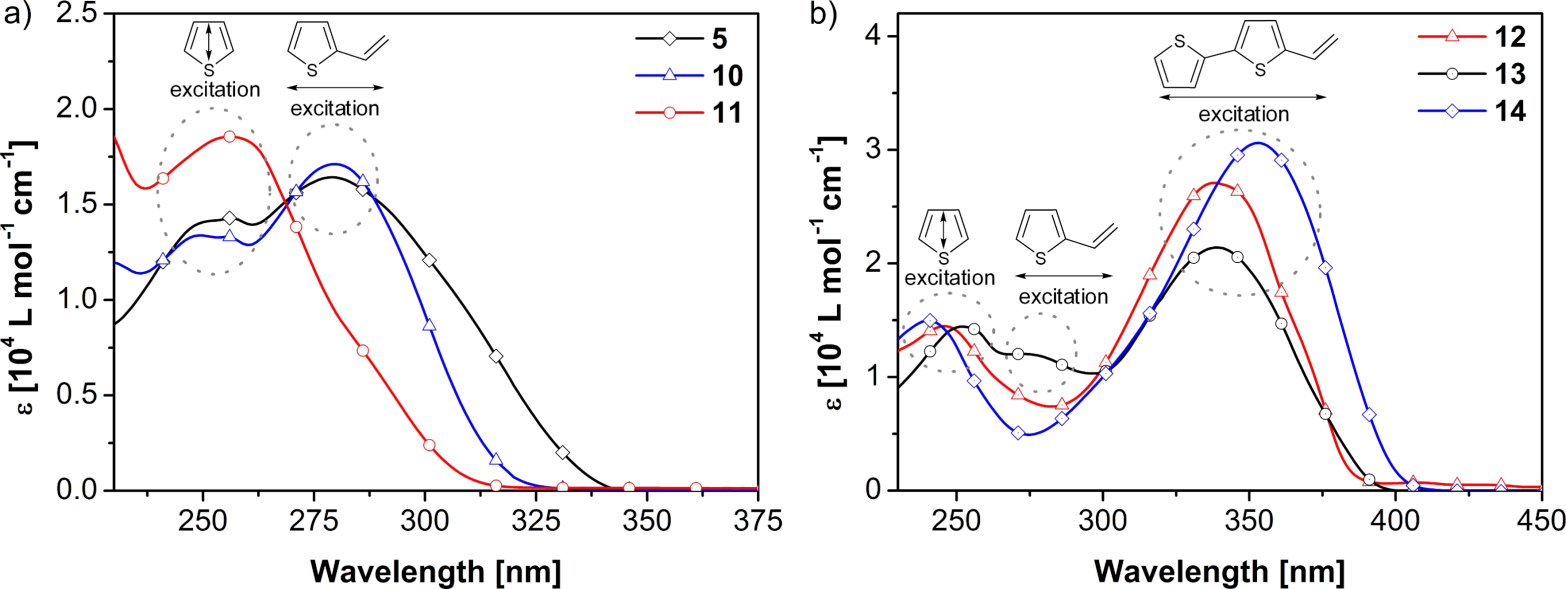
UV–vis spectra of **5**, **10** and **11** (a) and **12**–**14** (b) in dichloromethane ([c] = 5 × 10^−5^ M) at room temperature.

UV–vis absorption spectra of co-oligomers **5**, **6** and **10**–**14** revealed absorption bands of the individual subunits (Figure 1, Table 3). Thus, for triazole **5** an absorption band at 279 nm was observed, which is rather comparable to the absorption of 2-vinylthiophene (276 nm in ethanol) [38]. The second absorption band at 256 nm was assigned to the thiophene ring attached to the 1-position of the triazole. The evidence for intramolecular charge-transfer (ICT) [39] in **5** was investigated in several solvents with different dielectric constants: *n*-hexane (ε = 1.9), THF (ε = 7.6), methanol (ε = 32.6), and acetonitrile (ε = 37.5). Typically, no ICT was found in the described thiophene–triazole co-oligomers, but aggregate formation was observed in THF and *n*-hexane due to low solubility. For derivative **14** an absorption maximum at 353 nm was observed, which is red-shifted in comparison to that of **12** (λ_max_ = 337 nm) and **13** (λ_max_ = 339 nm). This band is not ascribed to individual bithienyl subunits, but to a weak conjugation through the triazole ring, caused by the donor–acceptor–donor system. Maarseveen et al. recently published synthesis and optical properties of 1,2,3-triazole containing co-polymers, suggesting that triazole rings interrupt conjugation and therefore no interaction of the various moieties of the polymer was observable [21].

**Table 3:** Spectroscopic and electrochemical characterization of **5**, **6** and **10**–**14**.

	λ^abs^ [nm]^a^	λ^em^_max_ [nm]	Φ [%]^b^	Stokes shift [cm^−1^]^c^	*E*^0^_ox_ [V]^d^

**5**	250, 256, *279*				1.15
**6**	*250*				1.45
**10**	248, 256, *279*				1.13
**11**	230, *256*				1.23
**12**	244, *337*	403	6	4 860	0.71
**13**	256, 273, *339*	414	10	5 344	0.89
**14**	240, *353*	424	14	4 744	0.69

^a^Maxima in italics (5 × 10^−5^ M in dichloromethane); ^b^quantum yields determined with respect to DPA [41]; ^c^Stokes shift is given for the 0→0^*^ transition (Δν = ν^abs^_max_ – ν^em^_max_), ^d^irreversible redox process, *E*^0^_ox_ determined at *I*_0_ = 0.855 × *I*_p_ [42].

Fluorescence spectra (10^−6^ M for **12** and **13**, 5 × 10^−7^ M for **14** in dichloromethane, Table 3) of investigated compounds **12**–**14** showed structured bands due to vibronic splitting. The red-shift of the emission maximum of **14** in comparison to **12** and **13** confirmed the facts assumed from UV–vis spectra that there should be weak electronic communication in the oligomers going through the 1,2,3-triazole ring. Fluorescence quantum yields of 6 to 14% were determined which are rather high for bithiophenes (1.8%) [40] and increased with increasing molecular size and conjugation. Obviously, the 1,2,3-triazole ring stabilizes the excited state by decreasing the probability of non-radiative deactivation.

Comparable to the optical properties, cyclic voltammetry of **5**, **6**, and **10**–**14** revealed redox transitions of the individual thiophene moieties (Table 3). Thus, cyclic voltammograms (CV) of **5** and **10** (5 × 10^−3^ M in dichloromethane/tetrabutylammonium hexafluorophosphate (TBAPF_6_, 0.1 M, 100 mV s^−1^) showed characteristic oxidation waves of the 2-vinylthiophene subunit at 1.15 V and 1.13 V vs Fc/Fc^+^, respectively. For co-oligomer **11**, the oxidation potential was significantly higher (1.23 V) and indicates formation of a radical cation localized on the thiophene subunits. In the negative regime, no reduction wave for the 1,2,3-triazole unit could be observed.

**Figure 2:**
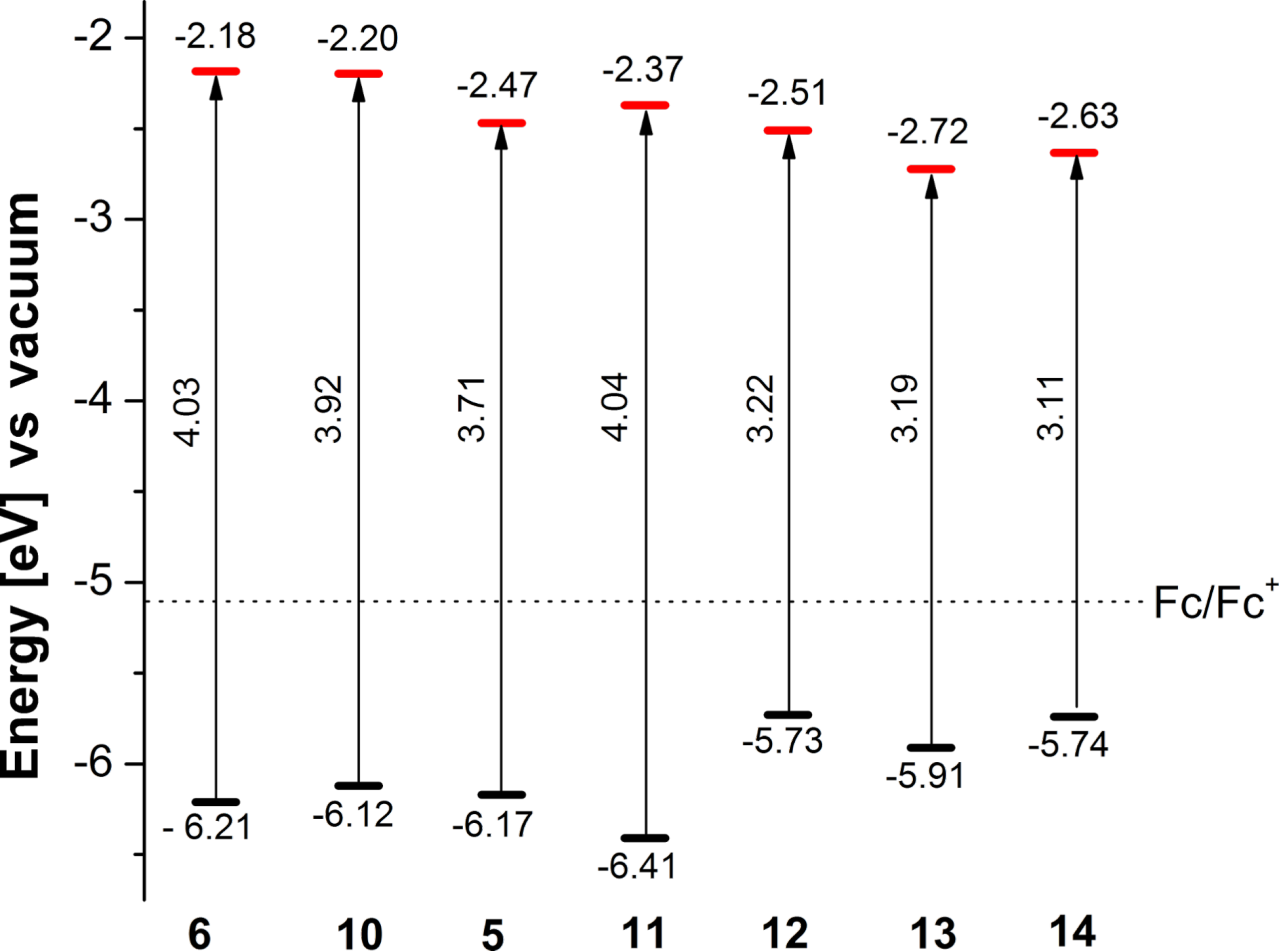
HOMO–LUMO energy level diagram for thiophene–triazole co-oligomers **5**, **6**, **10**–**14**.

From the optoelectronic data we deduced a HOMO–LUMO energy level diagram including band gaps for the novel donor–acceptor materials **5**, **6** and **10**–**14** (Figure 2). HOMO values were taken from the onset of oxidation and the internal reference Fc/Fc^+^ was set to −5.1 eV versus vacuum. The LUMO values were calculated by taking the optical band gaps into account, which were taken from the absorption onset at the lowest energy band. As a trend it can be seen that 3-thienyl derivative **11** has the largest band gap (4.04 eV), because the thiophenes are linked to the triazole by unfavorable β-connections. The gap successively decreases the more extended the conjugated π-system is and approaches 3.11 eV for α-connected bithienyl derivative **14**.

**Figure 3:**
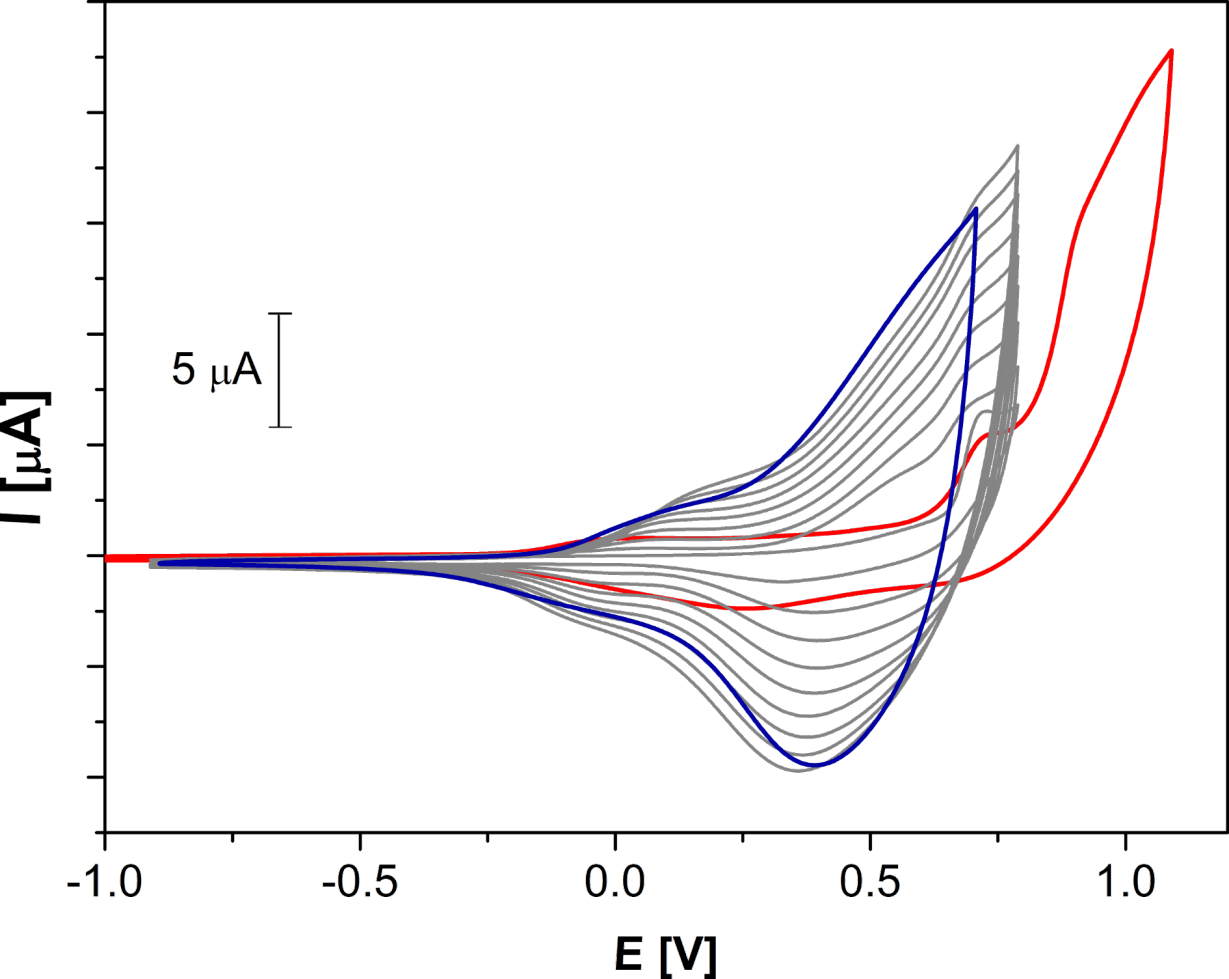
Cyclic voltammogram of bithienyl-triazole **14** in dichloromethane/TBAPF_6_ (0.1 M) versus Fc/Fc^+^ at 100 mV s^−1^ (red: monomer **14**, gray: during polymerization, blue: polymer film).

Oxidative oligo- and polymerization of derivatives **12**–**14** was carried out by potentiodynamic cycling in the appropriate potential range. The films, which were deposited on the platinum working electrode within 30 cycles, were investigated in monomer-free dichloromethane solution. The CVs indicated that only dimers of **12** and **13** were formed, which showed quasi-reversible oxidation waves at 0.28 V and 0.68 V versus Fc/Fc^+^, respectively, corresponding to a divinyl-quaterthiophene unit in **12** and a quaterthiophene in **13**. Electrochemical polymerization of **14** performed on a platinum working electrode in the range of −0.9 to 0.8 V versus Fc/Fc^+^ within 30 cycles is displayed in Figure 3 (monomer **14**: red, polymerization: gray, polymer film: blue). Because of similar oxidation potentials for both bithienyl subunits in **14** the formation of higher co-oligomers and co-polymers can be expected and the oxidation onset was located at −0.17 V. Therefore, comparable electronic structures in the oligo- and polymers are responsible for this redox behavior.

## Conclusion

In summary, a series of novel thiophene-1,2,3-triazole co-oligomers was synthesized in good to excellent yields by a three-component two-step procedure using copper-catalyzed [3 + 2]-Huisgen cycloaddition reactions. Spectroscopic and redox properties of selected donor–acceptor–donor derivatives were investigated, in which thiophene units act as donors and triazoles as acceptors. As a general result we find that weak electronic conjugation through the 1,2,3-triazole ring is operative.

## Experimental

### General information

All reactions were carried out under an inert atmosphere of argon. All chemicals were used as received without further purification unless otherwise specified. Thin-layer chromatography (TLC) was carried out on silica gel 60 F_254_ aluminium plates (Merck). Developed plates were dried and examined under a UV lamp. Preparative-column chromatography was carried out on glass columns of different diameters packed with silica gel, Merck 60 (40–63 µm). Gas chromatography (GC) was carried out using a Varian CP-3800 gas chromatograph. Helium 5.0 was used as the carrier gas; signals were examined by a flame-ionization detector (FID). Gas-chromatography–mass-spectrometry (GC–MS) measurements were executed with a Varian 3800. Helium 5.0 was used as the carrier gas; mass spectra were recorded on a Varian Saturn 2000. Ions were generated by electron impact (EI). Melting points were determined in a Büchi B-545 apparatus and are uncorrected. NMR spectra were recorded in CDCl_3_, DMSO-*d*_6_ or THF-*d*_8_ on a Bruker AMX 400 at 400 MHz (^1^H nuclei) and 100 MHz (^13^C nuclei), respectively. Chemical shifts are denoted in δ units (ppm) and are referenced to the solvent signal (7.26 ppm for CDCl_3_, 2.50 ppm for DMSO-*d*_6_ and 1.73 for THF-*d*_8_). The splitting patterns are designated as follows: s (singlet), d (doublet), t (triplet), m (multiplet). Mass spectra were measured at Finnigan MAT, SSQ 7000 by CI and Bruker Daltonics REFLEX III by MALDI-TOF. Elemental analysis for C, H and N were determined at Elementar Vario EL and for S at Carlo Erba 1104. High-resolution mass spectra were measured on a micrOTOF-Q 43 with electron spray ionization (ESI) and atmospheric-pressure chemical ionization (APCI). UV–vis spectra were taken on a Perkin-Elmer Lambda 19 in 1 cm cuvettes. Fluorescence spectra were measured with a Perkin-Elmer LS 55 in 1 cm cuvettes. Fluorescence quantum yields were determined with respect to 9,10-diphenylanthracene (DPA, Φ = 0.9 in dichloromethane) [43]. Cyclic voltammetry experiments were performed with a computer-controlled EG&G PAR 273 potentiostat in a three-electrode single-compartment cell (2 mL). The platinum working electrode consisted of a platinum wire sealed in a soft glass tube with a surface area of *A* = 0.785 mm^2^, which was polished down to 0.5 µm with Buehler polishing paste prior to use. The counter electrode consisted of a platinum wire and the reference electrode was an Ag/AgCl secondary electrode. All potentials were internally referenced to the ferrocene/ferricenium couple. For the measurements, the electroactive species were used in freshly distilled and degassed dichloromethane and 0.1 M tetrabutylammonium hexafluorophosphate (TBAPF_6_, Fluka), which was twice recrystallized from ethanol and dried under vacuum prior to use. 5'-Iodo-5,5''-bis(trimethylsilyl)-[2,2':3',2''terthiophene] and 2-[5,5"-bis(trimethylsilyl)-[2,2':3',2"-terthien]-5'-yl]ethynyl-1-trimethylsilane were synthesized according to the literature [44].

**3-Azidothiophene (2)**: In a mixture of ethanol (2.8 mL) and water (1.2 mL) 3-iodothiophene (**1**, 0.22 mL, 2 mmol), copper(I) iodide (38 mg, 0.2 mmol), sodium ascorbate (20 mg, 0.1 mmol), sodium azide (0.26 g, 4 mmol), and DMEDA (0.03 mL, 0.3 mmol) were dissolved and heated under refluxed under argon until TLC (silica/DCM) showed complete consumption of the starting material. After five hours the cooled brown mixture was diluted with water (10 mL) and ethyl acetate (10 mL). The aqueous phase was extracted with ethyl acetate (2 times, 10 mL). After the organic phases were washed with brine (15 mL) they were dried over sodium sulfate and evaporated to dryness in vacuum at room temperature. Because of the instability of the product on silica it was used without further purification. The NMR data was consistent with literature data [45]. ^1^H NMR (400 MHz, CDCl_3_) δ 6.79 (dd, *J* = 1.5 and 3.2 Hz, 1H), 6.82 (dd, *J* = 1.4 and 5.2 Hz, 1H), 7.30 (dd, *J* = 3.2 and 5.1 Hz, 1H).

### General procedure for the synthesis of 1,4-disubstituted 1*H*-1,2,3-triazoles

Halide (1 equiv) and terminal acetylene (1 equiv) were dissolved in an ethanol/water mixture (4 mL, 7:3). After the addition of sodium azide (2 equiv), sodium ascorbate (10 mol %), *N*,*N*’-dimethylethylenediamine (DMEDA, 20 mol %) and copper(I) iodide (10 mol %), the mixture was stirred in a closed Schlenk tube at 50 °C for about 15 hours. The cooled mixture was poured into 50 mL ice–water. If the product precipitated (method A) it was filtered off and washed with NH_4_OH (25 %) and water. The dried product was purified by column chromatography. The non-precipitating products (method B) were treated with 10 mL NH_4_OH (25 %). The aqueous solution was washed three times with 50 mL ethyl acetate. After the organic phase was dried over sodium sulfate, the crude product was concentrated at the rotary evaporator and purified on silica.

**1,4-Di(thien-2-yl)-1*H*-1,2,3-triazole (5):** 2-Iodothiophene (0.11 mL, 1 mmol) or 2-bromothiophene (0.10 mL, 1 mmol), 2-ethynylthiophene (0.11 mg, 1 mmol), sodium azide (130 mg, 2 mmol), copper(I) iodide (19 mg, 0.1 mmol), sodium ascorbate (20 mg, 0.1 mmol), DMEDA (20 µL, 0.2 mmol). Method A gave the white product in 0.161 g (0.69 mmol, 69%) from 2-iodothiophene and 0.186 g (0.80 mmol, 60%) from 2-bromothiophene. mp 125–126 °C (from toluene); ^1^H NMR (400 MHz, CDCl_3_) δ 7.06 (dd, *J* = 3.8 and 5.5 Hz, 1H), 7.11 (dd, *J* = 3.6 and 5.1 Hz, 1H), 7.25 (dd, *J* = 1.4 and 5.5 Hz, 1H), 7.29 (dd, *J* = 1.4 and 3.8 Hz, 1H), 7.35 (dd, *J* = 1.1 and 5.1 Hz, 1H), 7.48 (dd, *J* = 1,1 and 3.6 Hz, 1H), 8.00 (s, 1H); ^13^C NMR (100 MHz, CDCl_3_) δ 118.30, 118.35, 122.98, 124.80, 125.59, 126.29, 126.32, 132.04; CIMS *m*/*z*: (M + H) 234, (M − N_2_) 206; Anal. calcd for C_10_H_7_N_3_S_2_: C, 51.48; H, 3.02; N, 18.01; S, 27.49; found: C, 51.54; H, 3.13; N, 17.92; S, 26.87.

**1,4-Diphenyl-1*H*-1,2,3-triazole (6)**: Iodobenzene (0.11 mL, 1 mmol) or bromobenzene (0.11 mL, 1 mmol), phenylacetylene (0.11 mL, 1 mmol), sodium azide (0.13 g, 2 mmol), copper(I) iodide (19 mg, 0.1 mmol), sodium ascorbate (20 mg, 0.1 mmol), DMEDA (20 µL, 0.2 mmol). Method A gave triazole **6** in 0.199 g (0.90 mmol, 90%) from iodobenzene and in 0.923 g (0.42 mmol, 42%) from bromobenzene as a white solid. Changing the solvent to DMSO–water (9:1) gave **6** in 0.22 g (0.99 mol, 99%) from iodothiophene at 60 °C and bromobenzene at 95 °C, respectively. The analytical data correspond to literature data [21]. ^1^H NMR (400 MHz, CDCl_3_) δ 7.37 (m, 1H), 7.47 (m, 3H), 7.56 (m, 2H), 7.80 (m, 2H), 7.92 (m, 2H), 8.15 (s, 1H); ^13^C NMR (100 MHz, CDCl_3_) δ 120.6, 125.9, 128.4, 128.8, 128.9, 129.8.

**1-Phenyl-4-(thien-2-yl)-1*H*-1,2,3-triazole (7):** Iodobenzene (0.11 mL, 1 mmol), 2-ethynylthiophene (**4**, 0.11 g, 1 mmol), sodium azide (130 mg, 2 mmol), copper(I) iodide (19 mg, 0.1 mmol), sodium ascorbate (20 mg, 0.1 mmol), DMEDA (20 µL, 0.2 mmol). Method A gave the colorless product in 213.6 mg (940 µmol, 94%). mp 136–137 °C (from EE–*n*-hexane); ^1^H NMR (400 MHz, CDCl_3_) δ 7.12 (dd, *J* = 3.6 and 5.1 Hz, 1H), 7.35 (dd, *J* = 1.1 and 5.1 Hz, 1H), 7.47 (t, *J* = 7.3 Hz, 1H), 7.49 (dd, *J* = 1.1 and 3.3 Hz, 1H), 7.56 (t, *J* = 7.7 Hz, 2H), 7.78 (d, *J* = 7.4 Hz, 2H), 8.11 (s, 1H); ^13^C NMR (100 MHz, CDCl_3_) δ 117.06, 120.57, 124.56, 125.38, 127.71, 128.88, 129.80, 132.50, 136.93, 143.52; Anal. calcd for C_12_H_9_N_3_S: C, 63.41; H, 3.99; N, 18.49; S, 14.11; found: C, 63.40; H, 4.07; N, 18.44; S, 14.43; CIMS *m*/*z*: (M + H) 228, (M − N_2_) 199.

**4-Phenyl-1-(thien-3-yl)-1*H*-1,2,3-triazole (8**): 3-Iodothiophene (**1**, 0.13 mL, 1 mmol), phenylacetylene (0.11 mL, 1 mmol), sodium azide (130 mg, 2 mmol), copper(I) iodide (19 mg, 0.1 mmol), sodium ascorbate (20 mg, 0.1 mmol), DMEDA (20 µL, 0.2 mmol). Method A gave the pure off-white product in 0.186 g (0.82 mmol, 82%) from 3-iodothiophene and 0.145 g (0.64 mmol, 64%) from 3-bromothiophene. mp 168–169 °C (EE/*n*-hexane); ^1^H NMR (400 MHz, CDCl_3_) δ 7.37 (t, *J* = 7.3 Hz, 1H), 7.48 (t, *J* = 7.7 Hz, 2H), 7.49 (dd, *J* = 3.3 and 5.4 Hz, 1H), 7.53 (dd, *J* = 1.5 and 5.3 Hz, 1H), 7.61 (dd, *J* = 1.4 and 3.2 Hz, 1H), 7.90 (d, *J* = 7.1 Hz, 2H), 8.10 (s, 1H); ^13^C NMR (100 MHz, CDCl_3_) δ 114.14, 117.93, 120.85, 125.89, 127.29, 128.45, 128.92, 130.15, 148.01, 154.99; Anal. calcd for C_12_H_9_N_3_S: C, 63.41; H, 3.99; N, 18.49; S, 14.11; found: C, 63.51; H, 4.15; N, 18.46; S, 14.35; CIMS *m*/*z*: (M + H) 228, (M − N_2_) 199, (M − C_4_H_3_N_2_S) 116.

**4-Phenyl-1-(thien-2-yl)-1*H*-1,2,3-triazole (9**): 2-Iodothiophene (**3**, 0.11 mL, 1 mmol) or 2-bromothiophene (**3**, 0.10 mL, 1 mmol), phenylacetylene (0.11 mL, 1 mmol), sodium azide (130 mg, 2 mmol), copper(I) iodide (19 mg, 0.1 mmol), sodium ascorbate (20 mg, 0.1 mmol), DMEDA (20 µL, 0.2 mmol). According to method A the colorless product was obtained in 111 mg (0.49 mmol, 49%) from 2-iodothiophene and 120 mg (0.53 mmol, 53%) from 2-bromothiophene. mp 140–141 °C (from EE/*n*-hexane); ^1^H NMR (400 MHz, CDCl_3_) δ 7.07 (dd, *J* = 1.4 and 5.5 Hz, 1H), 7.30 (dd, *J* = 1.4 and 3.8 Hz, 1H), 7.39 (t, *J* = 7.4 Hz, 1H), 7.47 (t, *J* = 7.5 Hz, 2H), 7.89 (d, *J* = 7.1 Hz, 2H), 8.10 (s, 1H); ^13^C NMR (100 MHz, CDCl_3_) δ 118.18, 118.85, 122.83, 125.93, 126.28, 128.57, 128.93, 129.89, 138.43, 148.30; CIMS *m*/*z*: (M + H) 228, (M − N_2_) 199; Anal. calcd for C_12_H_9_N_3_S: C, 63.41; H, 3.99; N, 18.49; S, 14.11; found: C, 63.48; H, 4.03; N, 18.58; S, 13.92.

**4-(Thien-2-yl)-1-(thien-3-yl)-1*H*-1,2,3-triazole (10**): 3-Iodothiophene (**1**, 0.13 mL, 1 mmol) or 3-bromothiophene (0.10 mL, 1 mmol), 2-ethynylthiophene (**4**, 0.11 mg, 1 mmol), sodium azide (130 mg, 2 mmol), copper(I) iodide (19 mg, 0.1 mmol), sodium ascorbate (20 mg, 0.1 mmol), DMEDA (20 µL, 0.2 mmol). According to method A the off-white product was obtained in 0.231 g (0.99 mmol, 99%) from 3-iodothiophene and 0.11 g (0.47 mmol, 47%) from 3-bromothiophene. mp 157–158 °C (from EE/*n*-hexane); ^1^H NMR (400 MHz, CDCl_3_) δ 7.14 (dd, *J* = 3.6 and 5.1 Hz, 1H), 7.37 (dd, *J* = 1.1 and 5.1 Hz, 1H), 7.49 (dd, *J* = 1.1 and 3.5 Hz, 1H), 7.51 (dd, *J* = 2.5 and 5.6 Hz, 1H), 7.53 (dd, *J* = 1.6 and 5.3 Hz, 1H), 7.62 (dd, *J* = 1.6 and 3.1 Hz, 1H), 8.04 (s, 1H); ^13^C NMR (100 MHz, CDCl_3_) δ 100.00, 114.31, 117.41, 120.83, 124.61, 125.42, 127.35, 127.71; CIMS *m*/*z*: (M + H) 234, (M − N_2_) 206, (M − C_4_H_3_N_2_S) 122; HRMS–ESI (*m*/*z*): (M + Na) calcd for C_10_H_7_N_3_NaS_2_, 255.9974; found, 255.9981; (M + H) calcd for C_10_H_8_N_3_S_2_, 234.0154; found, 234.0157.

**1,4-Di(thien-3-yl)-1*H*-1,2,3-triazole (11)**: 3-Iodothiophene (**1**, 0.11 mL, 1 mmol), 3-ethynylthiophene (0.11 mg, 1 mmol), sodium azide (130 mg, 2 mmol), copper(I) iodide (19 mg, 0.1 mmol), sodium ascorbate (20 mg, 0.1 mmol), DMEDA (20 µL, 0.2 mmol). The product was obtained, according to method A, in 0.23 g (0.99 mmol, 99%) from 3-iodothiophene. mp 206–207 °C (from EE); ^1^H NMR (400 MHz, CDCl_3_) δ 7.42 (dd, *J* = 3.0 and 5.0 Hz, 1H), 7.48 (dd, *J* = 3.1 and 5.3 Hz, 1H), 7.51 (m, 2H), 7.60 (dd, *J* = 1.5 and 3.1 Hz, 1H), 7.76 (dd, *J* = 1.2 and 3.0 Hz, 1H), 8.01 (s, 1H); ^13^C NMR (100 MHz, CDCl_3_) δ 114.13, 117.73, 120.86, 121.63, 125.83, 126.51, 127.31; Anal. calcd for C_10_H_7_N_3_S_2_: C, 51.48; H, 3.02; N, 18.01; S, 27.49; found: C, 51.58; H, 3.11; N, 17.98; S, 27.73; CIMS *m*/*z*: (M + H) 234, (M − N_2_) 206, (M − C_4_H_3_N_2_S) 122.

**4-(2,2'-Bithien-5-yl)-1-(thien-2-yl)-1*H*-1,2,3-triazole (12**): 2-Iodothiophene (**3**, 0.11 mL, 1 mmol), 5-ethynyl-2,2'-bithiophene (190 mg, 1 mmol), sodium azide (130 mg, 2 mmol), copper(I) iodide (19 mg, 0.1 mmol), sodium ascorbate (20 mg, 0.1 mmol), DMEDA (20 µL, 0.2 mmol). The greenish-white product was obtained by method A in 195 mg (0.62 mmol, 62%). mp 159–160 °C dec (from toluene); ^1^H NMR (400 MHz, CDCl_3_) δ 7.04 (dd, *J* = 3.8 and 5.2 Hz, 1H), 7.06 (dd, *J* = 3.7 and 5.6 Hz, 1H), 7.17 (d, *J* = 3.8 Hz, 1H), 7.23 (dd, *J* = 1.3 and 3.7 Hz, 1H), 7.24 (dd, *J* = 1.1 and 2.1 Hz, 1H), 7.26 (m, 1H), 7.29 (dd, *J* = 1.4 and 3.8 Hz, 1H), 7.37 (d, *J* = 3.8 Hz, 1H), 8.00 (s, 1H); ^13^C NMR (100 MHz, CDCl_3_) δ 18.16, 118.39, 123.04, 124.08, 124.23, 124.78, 125.38, 126.33, 127.95, 130.67, 136.99, 137.62, 143.11; Anal. calcd for C_14_H_9_N_3_S_3_: C, 53.31; H, 2.88; N, 13.32; S, 30.49; found: C, 53.27; H, 3.00; N, 13.24; S, 30.32; CIMS *m*/*z*: (M + H) 316, (M − N_2_) 288.

**1-(2,2'-Bithien-5-yl)-4-(thien-2-yl)-1*H*-1,2,3-triazole (13**): 5-Iodo-2,2'-bithiophene (317 mg, 1 mmol), 2-ethynylthiophene (0.11 mg, 1 mmol), sodium azide (130 mg, 2 mmol), copper(I) iodide (19 mg, 0.1 mmol), sodium ascorbate (20 mg, 0.1 mmol), DMEDA (20 µL, 0.2 mmol). The pure greenish-white product was obtained by method A in 211 mg (0.67 mmol, 67%). mp 173–174 °C (from toluene); ^1^H NMR (400 MHz, CDCl_3_) δ 7.06 (dd, *J* = 3.6 and 5.1 Hz, 1H), 7.09 (d, *J* = 4.0 Hz, 1H), 7.12 (dd, *J* = 3.6 and 5.1 Hz, 1H), 7.18 (d, *J* = 4.0 Hz, 1H), 7.23 (dd, *J* = 1.1 and 3.6 Hz, 1H), 7.29 (dd, *J* = 1.1 and 5.1 Hz, 1H), 7.36 (dd, *J* = 1.1 and 5.1 Hz, 1H), 7.48 (dd, *J* = 1.1 and 3.6 Hz, 1H), 8.02 (s, 1H); ^13^C NMR (100 MHz, CDCl_3_) δ 117.79, 118.45, 122.29, 124.64, 124.87, 125.50, 125.67, 127.76, 128.05, 131.95, 135.89; CIMS *m*/*z*: (M^+^) 316, (M − N_2_) 288; HRMS–ESI (*m*/*z*): (M + Na) calcd for C_14_H_9_N_3_NaS_3_, 337.9851; found, 337.9847; (M + H) calcd for C_14_H_10_N_3_S_3_, 316.0031; found, 316.0026.

**1,4-Di(2,2'-bithien-5-yl)-1*H*-1,2,3-triazole (14)**: 5-Iodo-2,2'-bithiophene (0.92 g, 1 mmol), 5-ethynyl-2,2'-bithiophene (0.19 mg, 1 mmol), sodium azide (130 mg, 2 mmol), copper(I) iodide (19 mg, 0.1 mmol), sodium ascorbate (20 mg, 0.1 mmol), DMEDA (20 µL, 0.2 mmol). The pure orange product was obtained by method B in 131 mg (0.33 mmol, 33%). The extraction with ethyl acetate failed because of the low solubility of the product. It was therefore extracted with THF. mp 235–236 °C dec (from DCM/petrol ether); ^1^H NMR (400 MHz, DMSO-*d*_6_) δ 7.13 (dd, *J* = 3.6 and 5.0 Hz, 1H), 7.15 (dd, *J* = 3.6 and 5.1 Hz, 1H), 7.36 (d, *J* = 3.7 Hz, 1H), 7.37 (d, *J* = 3.2 Hz, 1H), 7.40 (dd, *J* = 0.9 and 3.5 Hz, 1H), 7.44 (dd, *J* = 1.0 and 3.6 Hz, 1H), 7.48 (d, *J* = 3.8 Hz, 1H), 7.54 (d, *J* = 4.0 Hz, 1H), 7.56 (dd, *J* = 0.9 and 5.1 Hz, 1H), 7.61 (dd, *J* = 1.0 and 5.1 Hz, 1H), 9.25 (s, 1H); ^13^C NMR (100 MHz, DMSO-*d*_6_) δ 115.79, 119.39, 119.96, 123.52, 124.97, 125.21, 125.60, 126.35, 126.97, 128.95, 129.02, 130.91, 134.14, 135.61, 135.90, 136.48, 136.86, 142.86; CIMS *m*/*z*: (M + H) 399, (M − N_2_) 371; HRMS–ESI (*m*/*z*): (M + Na) calcd for C_18_H_11_N_3_NaS_4_, 419.9728; found, 419.9716; (M + H) calcd for C_18_H_12_N_3_S_4_, 397.9909; found, 397.9907.

**1-(5-Methylthien-2-yl)-4-(thien-2-yl)-1*H*-1,2,3-triazole (15)**: 2-Methyl-5-iodothiophene (224 mg, 1 mmol), 2-ethynylthiophene (0.11 mg, 1 mmol), sodium azide (130 mg, 2 mmol), copper(I) iodide (19 mg, 0.1 mmol), sodium ascorbate (20 mg, 0.1 mmol), DMEDA (20 µL, 0.2 mmol). According to method A the pure white product was obtained in 168 mg (0.68 mmol, 68%). mp 112 °C dec (from methanol); ^1^H NMR (400 MHz, CDCl_3_) δ 2.52 (d, *J* = 1.0 Hz, 3H), 6.70 (dd, *J* = 1.1 and 3.7 Hz, 1H), 7.06 (dd, *J* = 3.7 Hz, 1H), 7.11 (dd, *J* = 3.6 and 5.1 Hz, 1H), 7.34 (dd, *J* = 1.1 and 5.1 Hz, 1H), 7.45 (dd, *J* = 1.1 and 3.6 Hz, 1H), 7.95 (s, 1H); ^13^C NMR (100 MHz, CDCl_3_) δ 15.41, 18.08, 118.41, 124.05, 124.68, 125.48, 127.70, 132.19, 144.63, 160.35, 160.56; Anal. calcd for C_11_H_9_N_3_S_2_: C, 53.42; H, 3.67; N, 16.99; found: C, 53.17; H, 3.81; N, 16.76; CIMS *m*/*z*: (M + H) 248, (M − N_2_) 220; HRMS–ESI (*m*/*z*): (M + Na) calcd for C_11_H_9_N_3_NaS_2_, 270.0130; found, 270.0139; (M + H) calcd for C_11_H_10_N_3_S_2_, 248.0311; found, 248.0323.

**1-(3-Methylthien-2-yl)-4-(thien-2-yl)-1*H*-1,2,3-triazole (16)**: 2-Iodo-3-methylthiophene (224 mg, 1 mmol), 2-ethynylthiophene (0.11 mg, 1 mmol), sodium azide (130 mg, 2 mmol), copper(I) iodide (19 mg, 0.1 mmol), sodium ascorbate (20 mg, 0.1 mmol), DMEDA (20 µL, 0.2 mmol). The product was obtained according to method B as an oil in 29.6 mg (0.12 mmol, 12%). ^1^H NMR (400 MHz, CDCl_3_) δ 2.26 (s, 3H), 6.91 (d, *J* = 5.6 Hz, 1H), 7.12 (dd, *J* = 3.6 and 5.1 Hz, 2H), 7.24 (d, *J* =5.5 Hz, 1H), 7.34 (dd, *J* = 1.1 and 5.1 Hz, 1H), 7.46 (dd, *J* = 1.0 and 3.6 Hz, 1H), 7.90 (s, 1H); ^13^C NMR (100 MHz, CDCl_3_) δ 13.47, 121.12, 123.27, 124.67, 125.50, 127.74, 129.20, 132.26, 132.91, 142.89; CIMS *m*/*z*: (M + H) 248, (M − N_2_) 219; HRMS–ESI (*m*/*z*): (M + Na) calcd for C_11_H_9_N_3_NaS_2_, 270.0130; found, 270.0124.

**Ethyl 5-(4-(thien-2-yl)-1*H*-1,2,3-triazol-1-yl)thiophene-2-carboxylate (17)**: Ethyl 2-iodo-5-thiophenecarboxylate (345 mg, 1 mmol), 2-ethynylthiophene (0.11 mg, 1 mmol), sodium azide (130 mg, 2 mmol), copper(I) iodide (19 mg, 0.1 mmol), sodium ascorbate (20 mg, 0.1 mmol), DMEDA (20 µL, 0.2 mmol). According to procedure B the product was obtained as a colorless solid in 186 mg (0.61 mmol, 61%). mp 135 °C dec (from DCM/petrol ether); ^1^H NMR (400 MHz, CDCl_3_) δ 1.40 (t, *J* = 7.1 Hz, 3H), 4.39 (q, *J* = 7.1 Hz, 2H), 7.13 (dd, *J* = 3.6 and 5.1 Hz, 1H), 7.28 (d, *J* = 4.1 Hz, 1H), 7.37 (dd, *J* = 1.1 and 5.1 Hz, 1H), 7.49 (dd, *J* = 1.1 and 3.6 Hz, 1H), 7.74 (d, *J* = 4.1 Hz, 1H), 8.05 (s, 1H); ^13^C NMR (100 MHz, CDCl_3_) δ 14.29, 61.75, 117.45, 117.50, 125.14, 125.94, 127.82, 130.61, 131.55, 132.44, 143.87, 161.49; Anal calcd for C_13_H_11_N_3_O_2_S_2_: C, 51.13; H, 3.63; N, 13.76; S, 21.00; found: C, 51.12; H, 3.71; N, 13.72; S, 21.07; CIMS *m*/*z*: (M + H) 306, (M − N_2_) 278, (M − C_2_H_5_N_2_) 250.

**Ethyl 2-(4-(thien-2-yl)-1*H*-1,2,3-triazol-1-yl)thiophene-3-carboxylate (18)**: Ethyl 2-iodo-3-thiophenecarboxylate (345 mg, 1 mmol), 2-ethynylthiophene (0.11 mg, 1 mmol), sodium azide (130 mg, 2 mmol), copper(I) iodide (19 mg, 0.1 mmol), sodium ascorbate (20 mg, 0.1 mmol), DMEDA (20 µL, 0.2 mmol). According to procedure B no product could be isolated.

**1-[5,5''-Bis(trimethylsilyl)-[2,2':3',2''-terthien]-5'-yl]-4-(thien-2-yl)-1*H*-1,2,3-triazole (19):** 5'-Iodo-5,5"-bis(trimethylsilyl)-[2,2':3',2"-terthiophene] (259 mg, 0.5 mmol), 2-ethynylthiophene (54 mg, 0.5 mmol), sodium azide (65 mg, 1 mmol), copper(I) iodide (10 mg, 50 µmol), sodium ascorbate (10 mg, 50 µmol), DMEDA (10 µL, 0.1 mmol). By method B the product was obtained in 111 mg (0.205 mmol, 41%) as yellowish needles. mp 119–120 °C (from petrol ether); ^1^H NMR (400 MHz, CDCl_3_) δ 0.32 (2 × s, 18H), 7.11 (dd, *J* = 3.7 and 5.2 Hz, 1H), 7.13–7.16 (m, 3H), 7.22 (d, *J* = 3.4 Hz, 1H), 7.34 (s, 1H), 7.35 (dd, *J* = 1.0 and 5.1 Hz, 1H), 7.48 (dd, *J* = 1.0 and 3.5 Hz, 1H), 8.03 (s, 1H); ^13^C NMR (100 MHz, CDCl_3_) δ −0.15, −0.1, 117.52, 120.04, 124.89, 125.68, 127.75, 128.21, 128.67, 129.62, 130.77, 131.91, 134.21, 135.88, 138.46, 141.37, 141.55, 143.18, 143.45, 143.53, 145.41; HRMS–ESI (*m*/*z*): (M + Na) calcd for C_24_H_27_N_3_NaS_4_Si_2_, 564.0519; found, 564.0513.

**4-[5,5''-Bis(trimethylsilyl)-[2,2':3',2''-terthien]-5'-yl]-1-(thien-2-yl)-1*H*-1,2,3-triazole (20)**: 2-Iodothiophene (0.03 mL, 0.25 mmol), 2-[5,5"-bis(trimethylsilyl)-[2,2':3',2"-terthien]-5'-yl]ethynyl-1-trimethylsilane (125 mg, 0.3 mmol), sodium azide (35 mg, 0.5 mmol), copper(I) iodide (5 mg, 25 µmol), sodium ascorbate (5 mg, 25 µmol), DMEDA (5 µL, 50 µmol). By method A the product was obtained as yellow solid in 124 mg (0.23 mmol, 92%). mp 129–130 °C (from petrol ether); ^1^H NMR (400 MHz, CDCl_3_) δ 0.32 (2 × s, 18H), 7.07 (dd, *J* = 3.8 and 5.4 Hz, 1H), 7.12–7.15 (m, 3H), 7.21 (d, *J* = 3.5 Hz, 1H), 7.25 (m, 1H), 7.29 (dd, *J* = 1.3 and 3.8 Hz, 1H), 7.52 (s, 1H), 8.02 (s, 1H); ^13^C NMR (100 MHz, CDCl_3_) δ −0.09, −0.04, 118.29, 118.40, 123.06, 126.34, 127.47, 127.86, 128.94, 130.28, 131.73, 132.29, 134.20, 138.13, 139.79, 140.87, 142.30, 142.50, 142.76; CIMS *m*/*z*: (M + H) 542, (M − N_2_) 513, (M − Si(CH_3_)_3_) 470, (M − N_2_Si(CH_3_)_3_) 441, (M − 2Si(CH_3_)_3_) 398; HRMS–ESI (*m*/*z*): (M + Na) calcd. for C_24_H_27_N_3_NaS_4_Si_2_, 564.0519; found, 564.0514.

**1,4-Phenylene-bis(4'-(thien-2''-yl)-1'*H*-1',2',3'-triazol-1'-yl) (21)**: 1,4-Diiodobenzene (330 mg, 1 mmol) or 1,4-dibromobenzene (236 mg, 1 mmol), 2-ethynylthiophene (0.22 mg, 2 mmol), sodium azide (260 mg, 4 mmol), cupric sulfate pentahydrate (50 mg, 0.2 mmol), sodium ascorbate (40 mg, 0.2 mmol), DMEDA (40 µL, 0.4 mmol). The reaction was performed in DMSO–water (9:1). 1,4-Diiodobenzene yielded in 372 mg (0.99 mmol, 99%) at 50 °C, from 1,4-dibromobenzene the disubstituted product was obtained in 368.5 mg (0.98 mmol, 98%) at 95 °C. mp 310 °C dec (from toluene); ^1^H NMR (400 MHz, THF-*d*_8_) δ 7.09 (dd, *J* = 3.6 and 5.1 Hz, 2H), 7.41 (dd, *J* = 1.1 and 5.1 Hz, 2H), 7.50 (dd, *J* = 1.1 and 3.6 Hz, 2H), 8.15 (s, 4H), 8.77 (s, 2H); ^13^C NMR (100 MHz, DMSO-*d*_6_) δ 100.00, 119.47, 121.89, 125.26, 126.59, 128.56, 132.63, 136.68, 143.41; CIMS *m*/*z*: (M + H) 377, (M − N_2_) 349, (M − N_4_) 320; HRMS–ESI (*m*/*z*): (M + Na) calcd for C_18_H_12_N_6_NaS_2_, 399.0457; found, 399.0452.
